# Biophysical interplay between extracellular matrix remodeling and hypoxia signaling in regulating cancer metastasis

**DOI:** 10.3389/fcell.2024.1335636

**Published:** 2024-03-13

**Authors:** Sun-Ah Lee, Gi-Ju Cho, Doyoung Kim, Dong-Hwee Kim

**Affiliations:** ^1^ KU-KIST Graduate School of Converging Science and Technology, Korea University, Seoul, Republic of Korea; ^2^ Department of Integrative Energy Engineering, College of Engineering, Korea University, Seoul, Republic of Korea; ^3^ Biomedical Research Center, Korea Institute of Science and Technology, Seoul, Republic of Korea

**Keywords:** tumor-microenvironment, ECM remodeling, hypoxia, cancer metastasis, mechano-signaling, mechano-regulation, cancer biophysics

## Abstract

Mechanical properties of the tumor microenvironment play a critical role in cancer progression by activation of cancer mechano-responses. The biophysical interactions between cancer cells and their dynamic microenvironment are attributed to force-dependent alterations in molecular pathways that trigger the structural reorganization of intracellular organelles and their associated genetic modifications. Recent studies underscore the role of oxygen concentration in cancer metastasis. Suppressed oxygen levels promote the development of invasive phenotypes and aggressive proliferation of cancer cells, accompanied by remodeling of tumor microenvironment encompassing the modulation of physical settings of extracellular matrix. This review summarizes the role of biophysical interactions between cancer cells and their surroundings in determining cancer progression. Biophysical interpretation of the tumor microenvironment and cancer progression could provide further insights into the development of novel biomedical technologies for therapeutic cancer treatment.

## 1 Introduction

Cancer characterized by uncontrollable cell growth and proliferation, is a prominent cause of human mortality, posing an enduring challenge to modern medicine. Cancer research has traditionally focused on understanding the underlying cancer development, providing valuable insights into the molecular basis of the disease. However, recent studies highlight the crucial role of the tumor microenvironment (TME) hypoxic condition. Furthermore, physical properties such as matrix viscoelasticity, extracellular fluid viscosity, ligand density, osmotic pressure, and oxygen concentration have been identified as critical determinants of cancer growth, progression, and metastasis.

This study focused on two critical biophysical elements, hypoxia and dimensions, as integral components of the TME that profoundly regulate cancer cell proliferation. Understanding their individual and synergistic effects on tumors is crucial for understanding the properties of cancer cells. Cells in three-dimensional (3D) environments encounter different physical cues, such as cell-cell contacts, extracellular matrix (ECM) stiffness, and spatial constraints, which can alter their migratory characteristics. It is important to understand that conclusions drawn from two-dimensional (2D) studies may not consistently mirror the behavior and responses of cells within the intricate and physiologically relevant 3D microenvironments encountered in living organisms (Wu et al., 2014; Luzhansky et al., 2018; Baskaran et al., 2020).

Cells within the 3D TME show complex interactions with various physical properties of ECM. Moreover, it is necessary to consider ECM arrangement, organization, and composition to better interpret dimension-dependent altered cell migration. Furthermore, studies have addressed the structural elements in the context of cancer, including dimensions, pore size, porosity, fiber thickness, and fiber orientation (Murphy and O’Brien, 2010).

Hypoxia, a reduced oxygen state in the TME, is a hallmark of many solid tumors and is associated with aggressive cancer cell behaviors. It induces adaptive responses in cancer cells through angiogenesis and altered metabolism (Haage and Schneider, 2015; Wei et al., 2015; Zhao D. et al., 2018). In addition, hypoxia-controlled enzymes can alter the composition of the ECM and cytoskeletal organization (Weidemann et al., 2013). Hypoxia-inducible factors (HIFs) are crucial transcription factors for tumor growth and metastasis and play a central role in regulating various aspects of cell metabolism (Acerbi et al., 2015). Understanding how cancer cells respond to hypoxia and the subsequent effects on cell proliferation is essential for developing targeted therapies (Liverani et al., 2019).

Here, we investigated the effect of cancer-specific mechanosensitive proteins and signaling molecules of various cancer types. In several cases, increased ECM stiffness enhances immunosuppression by activating immune-associated marker proteins. In particular, programmed death-ligand 1 (PD-L1) and transforming growth factor- β (TGF-β) are regulated in some types of cancer due to changes in ECM stiffness (Zhang et al., 2023). Moreover, studies have shown that cancer cells undergoing specific changes, such as cell morphology, cytoskeletal architecture, adhesion clustering, and signaling pathways, may develop a heightened resistance to shear stress (Hann et al., 2022), enabling them to endure the mechanical pressure encountered during metastasis. Cancer cells can potentially amplify their survival capacity and persistence in circulation, facilitating the establishment of secondary tumors in distant organs.

This study comprehensively examines the physical elements underlying the structure and dynamics of the TME in the 2D and 3D contexts. We also explored how the interplay between oxygen concentration and ECM remodeling determines cell migration. Furthermore, various immune responses and malignancy profiles of different cancer types in response to external mechanical stimuli highlight the importance of understanding the diverse effects of hypoxia and the potential therapeutic prospects of targeting biophysical interactions to impede cancer progression (Osuna de la Peña et al., 2021).

## 2 Biophysical interpretation of tumor microenvironment (TME)

### 2.1 Physical aspects of structure and dynamics of TME

#### 2.1.1 Cellular responses in 3D microenvironment

The cell, through its membrane receptors, governs a broad spectrum of cellular functions and coordinates the intricate development of multicellular organisms by engaging in mechanical interactions with other cells and the ECM. Integrins, in particular, are recognized as the primary cell transmembrane receptors firmly anchored to the cytoskeleton. These receptors play a crucial role in transmitting both biochemical and mechanical signals between cells and the ECM (Chen et al., 2017). Integrin-mediated mechanotransduction operates across diverse dimensions, guiding biophysical cellular responses in intricate ways. Whether in two-dimensional (2D) or three-dimensional (3D) environments, these processes play a crucial role in translating mechanical signals into biochemical responses, influencing various aspects of cell behavior (Harunaga and Yamada, 2011). Specifically, gaining insight into the mechanotransductive role of integrins across various dimensions is crucial for a comprehensive understanding of the intricate cellular dynamics associated with processes such as migration, proliferation, and differentiation. Beyond integrins, it is imperative to explore how cellular mechanosensing occurs through adhesion complexes within 3D microenvironments and elucidate the potential variations and differences in these mechanisms compared to interactions on 2D substrates.

**FIGURE 1:**
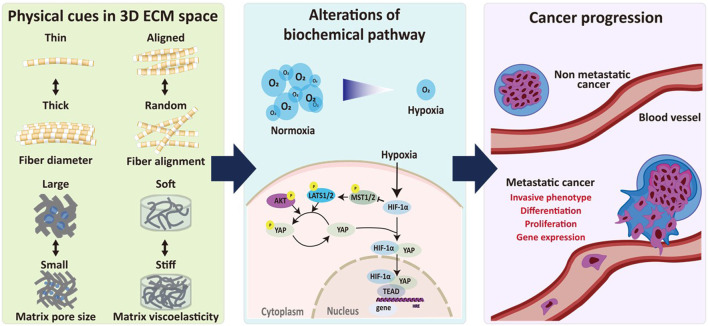
Biophysical interplay between ECM remodeling and hypoxia signaling in regulating cancer progression. Biophysical relationship between ECM remodeling and hypoxia-induced signaling pathways plays an important role in regulating cancer progression. Physical cues in 3D ECM including fiber diameter and alignment as well as pore size and viscoelasticity of the ECM regulates hypoxia-induced biochemical pathways, resulting in progression of metastatic alteration.

Understanding cancer cell behavior in a 3D context facilitates a more realistic evaluation of cancer metastasis in cellular level, including proliferation, migration, and invasion, and provides valuable insights into the disparities in cell responses in microenvironments of various dimensions and the phenomenon of cell response mismatch. ECM remodeling and hypoxia signaling interact to drive cancer progression by altering the physical properties of the ECM (Figure 1). In 3D, cells interact with the surrounding ECM more intricately than in 2D settings. The composition, density, and spatial arrangement of the ECM play critical roles in modulating cell behaviors such as proliferation and differentiation (Tang et al., 2014; Guetta-Terrier et al., 2015; Yuan et al., 2023). Studies on cell migration on 1D microprinted lines, suspended fibers, and 2D surfaces demonstrate that cells generally migrate faster and have higher persistence on suspended nanofibers, which is more relevant for mimicking 3D cell migration than microprinted 1D lines on 2D surfaces. Researchers found that Arp2/3 mediates actin polymerization-derived fin-like protrusion waves. These wave-like structures are frequently observed when cells migrate to suspended fibers. In addition, these waves are generated through the balanced activation of diverse cytoskeleton-related pathways, including the Rac1/N-WASP/Arp2/3 and Rho/formins pathways (Guetta-Terrier et al., 2015). These findings indicate that attempts to replicate 3D migration dynamics by employing cell migration on microprinted 1D lines on 2D surfaces may not comprehensively emulate the authentic behavior exhibited by cells during migration.

**FIGURE 2:**
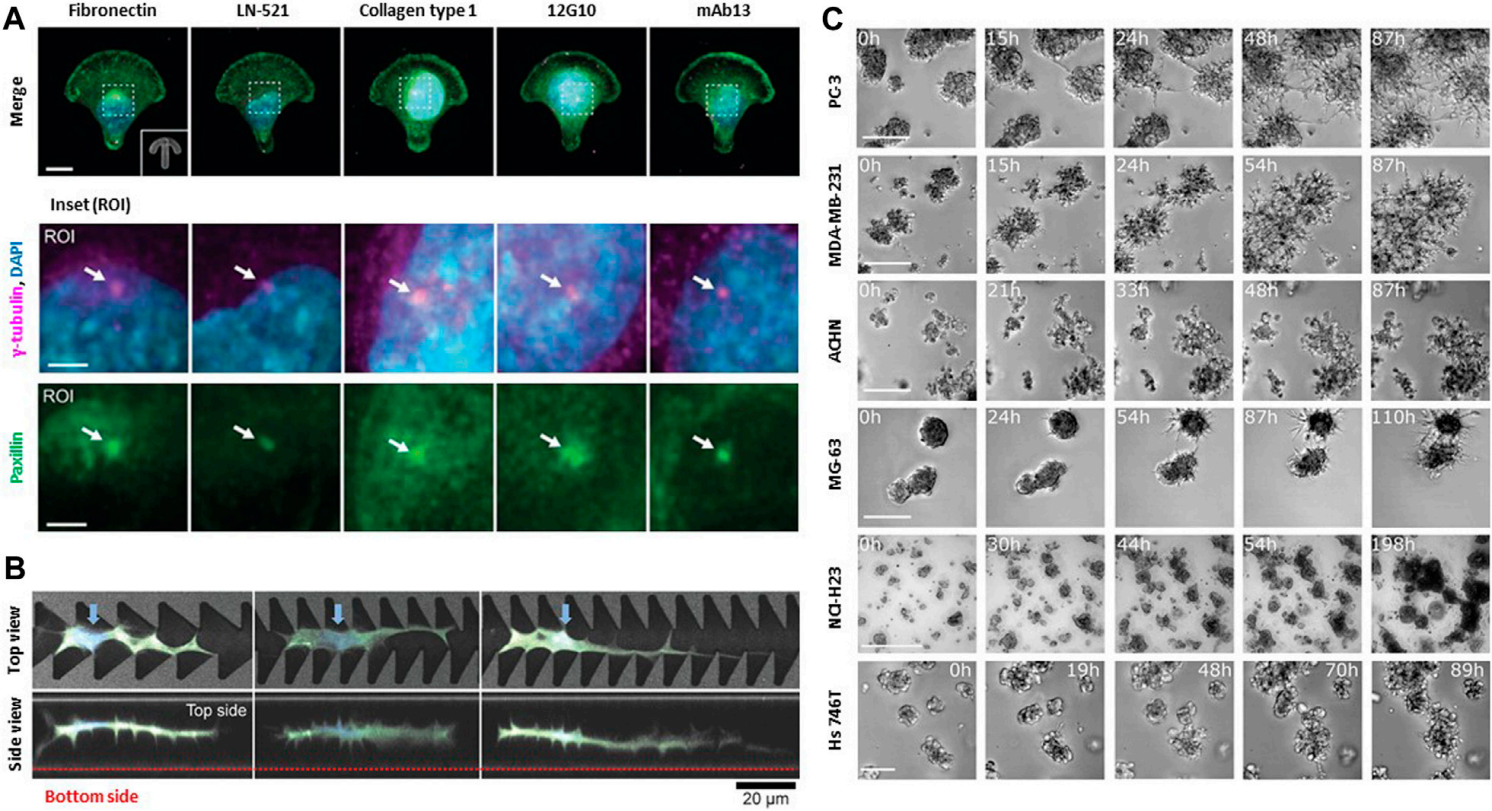
Biophysical interactions between cell and extracellular environments. **(A)** Specific cell-matrix interactions are due to different integrin ligands and FAK activity. Both influence front-rear polarization and organelle rearrangement on anisotropic micropatterns. Representative immunofluorescence images of U-251MG cells on crossbow-shaped micropatterns coated with diverse ECM components or integrin β1-targeting monoclonal antibodies. ROIs depict centrosomes (white arrows), visualized by staining of γ-tubulin and paxillin. Front-rear polarization was evaluated based on the orientation of the perinuclear centrosome, indicated by γ-tubulin or centrin-2, relative to the nucleus. Scale bar: 10 μm and 3 μm (ROI). Copyright obtained from reference [Aleksi Isomursu et al., Small methods (2023)]. **(B)** NIH3T3 cells adapts their surrounding environments to navigate through restrictive space. Top views and side views of confocal laser scanning microscopy images of cells extending protrusion along the sharp edges of the portions of the grooves on the micropatterns. Blue arrows indicate the cell nuclei. NIH3T3 cells can navigate complex, confined 3D spaces via morphological adaptations Scale bar: 20 μm. Copyright obtained from reference [Yusuke Shimizu et al., Advanced Biosystems (2020)]. **(C)** Collective cells in spheroids move directionally towards each other. All aggregating cell lines including a human prostate cancer cell line (PC-3), epithelial human breast cancer cell line (MDA-MB-231), primary renal cancer cell line (ACHN), osteosarcoma cell line (MG-63), lung cancer cell line (NCI-H23), gastric cancer cell line (Hs746T) recapitulate the same behavior observed in aggregation assays either starting from single cells or from spheroids. The fraction of aggregating cells was calculated by manually screening all cells appearing in the first frame. Scale bar: 500 μm. Copyright obtained from reference [Miriam Palmiero et al., Molecular Oncology (2020)].

The impact of these dimensions extends beyond just migration. This also extends to morphological and phenotypic variations (Figure 2A) (Isomursu et al., 2023). Cells tend to flatten and concurrently lose their distinct phenotypes (Kapalczynska et al., 2018). Although 2D surfaces have been extensively studied and have provide valuable insights, the behavior of cells in 2D environments may not directly translate to their behavior in 3D microenvironments, which better resembles the actual *in vivo* conditions. For cells on micro-contact printed lines, the leading edge of the cell is restricted by the width of the line and short protrusion-retraction cycles in the zone responsible for leading-edge advancement (Doyle et al., 2009). Notably, cells on suspended fiber, which is more relevant to *in vivo*-like 3D microenvironments, show lamellipodia-like actin structure under the Rac1-Arp2/3 signaling cascade, forming fin-like protrusions at the focal adhesion site and the lateral diffusion on fins is the leading cause of edge extension and cell migration (Guetta-Terrier et al., 2015). In a 2D environment, cells interact primarily with the substrate underneath them, which lacks the complex spatial and mechanical cues present in a 3D microenvironment. The ECM primarily exists as a 3D network of fibers, proteins, and other components that provide various physical and chemical cues to cells, such as substrate or matrix stiffness in and out of tissues/organs, topology, geographic features, compressive force by heart-beat, and *in vivo* shear stress due to blood flow.

In confined 3D microenvironments, cells encounter additional challenges compared with migration on 2D surfaces leading to diverse cellular responses. For example, cellular activities such as adhesion, proliferation, migration, and infiltration are regulated by the matrix pore size (Murphy and O’Brien, 2010). Cancer cell morphology and invasiveness are regulated by the diameter of the ECM fiber (Sapudom et al., 2015). ECM alignment dictates the polarization of focal adhesions and protrusions by localizing Rac activity, which controls tumor invasiveness and modulates collective ECM remodeling through its distinct pushing/pulling deformation within diverse tumor geometry (Wang et al., 2018; Kim et al., 2020). Cells require multiple adaptation mechanism to navigate through a more complex and restrictive in 3D microenvironment compared to 2D migration (Figure 2B) (Sunami et al., 2020). 3D cell migration involves interactions with neighboring cells, as demonstrated via photothermal ablation of the 3D wound healing model, inhomogeneities of the 3D ECM matrix (e.g., composition and structural features), and various signaling pathways that make it a more complex process than in 2D environments (Figure 2C) (Devreotes and Horwitz, 2015; Xiao et al., 2019; Hayn et al., 2020; Palmiero et al., 2023).

#### 2.1.2 Physical characteristics of extracellular matrix

Structural properties of TME are determined by the arrangement, organization, and composition of the protein components within the ECM, and they collectively establish the fundamental framework or architecture of the ECM. Cells exhibit the capability to align their cytoskeleton along the polarity axis and migrate directionally in response to spatial gradients of ECM, which is collectively termed topotaxis. Observed both on the microscale and nanoscale, topotaxis arose from the material characteristics of cells and the surrounding ECM implies the cellular sensitivity to topographical gradients of the ECM (Park et al., 2018). The invasive potential of numerous cancers might be dependent on broad topotactic responses, presenting a potentially compelling mechanism for regulating invasive and metastatic behaviors. The directional response of topotaxis is associated with the mechanical balance between cell and ECM and tightly regulated by signaling pathways such as phosphoinositide 3-kinase (PI3K)/protein kinase B (AKT) pathway and Rho-kinase (ROCK)/Myosin light chain kinase (MLCK). This balance, in turn, regulates the effective stiffness of the cortical cytoskeleton and the plasma membrane, determining the directionality of topotaxis (Park J. et al., 2016).

Porosity and permeability regulate tumor invasion and metastasis, and cancer cells interact with the surrounding ECM structures. Decoupling the effects of ECM density and porosity can be challenging because of their inherent coupling. Altering the ECM porosity independently of the ECM density presents a crucial challenge because changes in density induce changes in porosity. One potential approach to achieve this decoupling is to employ nanofibers and apply compression (Huang et al., 2019). This approach allows for the independent manipulation of ECM porosity and density by controlling the interaction between interfiber bondings that is essential for adjusting the mechanical characteristics of the bulk material during the polymerization process (Sapudom et al., 2019). Increasing ECM alignment also allows cells to adopt an elongated uniaxial morphology and migrate at enhanced speed and persistence. Indeed, the organization of focal adhesions was tightly correlated with the migration speed, with cells with the most aligned adhesions displaying the fastest migration (Wang et al., 2018). The bending stiffness of fibrils, determined by their resistance to deformation, is closely linked to the diameter of the ECM fibrils (Sapudom et al., 2019). In case that the fibril diameter increases, the bending stiffness also increases, and *vice versa*. Evidences shows that cancer cell invasion is controlled by the fibril diameter and pore size of 3D fibrous networks (Sapudom et al., 2015; Sapudom et al., 2019).

Viscoelasticity has been found to be a near-universal characteristic of living tissues and ECMs (Chaudhuri et al., 2020). Viscoelastic materials deform in a time-dependent manner in response to the application of an external stress or load (Chaudhuri et al., 2020). Plasticity refers to the ability of cancer cells to modify their physiological characteristics, allowing them to survive in hostile microenvironments and resist clinical therapies (Poltavets et al., 2018). Enhanced matrix plasticity promotes the spreading and motility of MDA-MB-231 cells (human breast cancer cell line) independent of proteases and extends invadopodia protrusions into IPN 3D matrices (Wisdom et al., 2018). A rigid matrix hindered the protrusion of individual MDA-MB-231 cells, and in matrices with low plasticity, the dislocation of cortactin (an actin polymerization-related protein) and F-actin implied disrupted actin polymerization (Wisdom et al., 2020).

Various cellular behaviors are critically governed by the distinctive properties of the ECM, including matrix density, porosity, and rigidity (Artym et al., 2015; Wisdom et al., 2018; Wisdom et al., 2020; Mierke, 2021; Wei et al., 2023). Investigation of mechanisms underlying diverse cancer cell responses to the surrounding microenvironment is essential for understanding cancer mechanobiology (Lee et al., 2019). Recent advances *in vitro* TME modeling unveil interactions between cell and microenvironments. Modulation of oxygen concentration mimics the hypoxic TME to reproduce HIF stabilization and nuclear translocation effects *in vitro*. Hypoxia governs the composition, deposition, post-translational modifications, and rearrangement of the ECM. Severe hypoxic tumor cores with cells expressing HIF-1α are localized to the nuclei in the larger tumors, whereas weaker and more diffused HIF-1α signals are present throughout the smaller tumors. These results imply that oxygen-gradients play a key molecular regulator of tumor progression (Table 1).

**TABLE 1:** Alterations in cancer and TME with increased HIF-1α in hypoxia.

Cancer and TME response	Molecular regulator	Alteration in cancer and TME	References
Extracellular matrix remodeling	P4HA1 and P4HA2	Collagen deposition	Morimoto et al. (2021)
	PLOD2	ECM stiffening and collagen fiber alignment	Morimoto et al. (2021)
	MMP2	ECM remodeling	Deschene et al. (2012)
Angiogenesis	Eukaryotic translation elongation factor 1 alpha 2 (EEF1A2)	Microvascular density↑	Lewis et al. (2016)
	VEGF	Microvascular density↑	Bos et al. (2001)
Actin polymerization	Ras homolog family member A (RhoA)/Rho associated coiled coil containing protein kinase 1 (ROCK1)	Actomyosin contractility↑	Semenza (2016)
	Focal adhesion kinase (FAK)	Cell motility ↑	Semenza (2016)

### 2.2 *In vitro* TME mimicry in hypoxia

HIF-1α-dependent collagen remodeling in sarcoma cells was recapitulated by Young’s modulus of hypoxic and non-hypoxic hydrogels compared with day 0 and day 3 of culture which were significantly decreased in cell-laden hydrogels under hypoxic conditions (Lewis et al., 2016). Furthermore, collagen deposition and reverse transcription polymerase chain reaction (RT-PCR) analysis of collagen modification genes collagen 1 A1 (COL1A1), lysl oxidase (LOX), and Procollagen-Lysine,2-Oxoglutarate 5-Dioxygenase 2 (PLOD2) significantly increased under hypoxia, indicating that hypoxic gradients promote tumor cell migration (Eisinger-Mathason et al., 2013; Lewis et al., 2016). Systemic sclerosis (SSc) is an uncommon long-term condition of unknown origin marked by widespread fibrosis and vascular irregularities regulating the skin, joints, and internal organs (Denton and Khanna, 2017). Inadequate angiogenesis leading to tissue ischemia and build-up of the ECM are characteristic features of SSc (Manetti et al., 2010). Hypoxia directly promotes fibrosis in individuals with SSc by enhancing the release of key ECM proteins (Distler et al., 2007).

Hypoxic stimuli significantly enhanced the proliferation of equine dermal fibroblasts (EDFs). The hypoxia-mimicking substance CoCl2 increased COL1A1 expression and reduced matrix metalloproteinases2 (MMP2) expression, indicating increased ECM synthesis and reduced turnover (Deschene et al., 2012). Both effects were suppressed by echinomycin that inhibits the activity of HIF-1, revealing its reliance on HIF mediated transcriptional regulation (Yonekura et al., 2013). Because hypoxia governs the composition, deposition, posttranslational modifications, and rearrangement of the ECM, hypoxia-induced vascular remodeling is intricately controlled by adjusting the activities of ECM-modifying enzymes, which subsequently regulate the availability of matricellular proteins and growth factors (Karavasili and Koolwijk, 2022).

Hypoxia prompts angiogenesis by increasing expression of growth factors, particularly vascular endothelial growth factor (VEGF), which guides endothelial cells to form tip cells and drives capillary sprout growth. (Berra et al., 2000). Hypoxia-induced basement membrane deposition and mechanical ECM signals may coordinate vessel sprouting via the VEGF and Notch pathways (Germain et al., 2010). In breast cancer, hypoxic signaling activates various mechanisms directly involved in ECM remodeling, leading to increased aggressiveness (Semenza, 2016). They synergistically boost aerobic glycolysis by increasing glucose transport and glycolytic enzyme levels and regulating intracellular pH (Dekker et al., 2022). Moreover, HIF-1 activates genes encoding collagen prolyl 4-hydroxylase subunit α-1 and prolyl 4-hydroxylase subunit α-2 (P4HA1 and P4HA2) and PLOD2 hydroxylases. P4HA1 and P4HA2 are essential for collagen deposition, whereas PLOD2 is crucial for ECM stiffening and collagen fiber alignment. Together, these enzymes, mediated by HIF-1, drive alterations in ECM composition, alignment, and mechanical properties in response to hypoxia. HIF-1-dependent ECM remodeling by hypoxic fibroblasts leads to changes in breast cancer cell morphology, adhesion, and motility, thereby facilitating invasion and metastasis (Morimoto et al., 2021). In summary, replicating the TME using *in vitro* models offers numerous advantages. The ability to emulate and observe cell behavior in response to oxygen gradients within a 3D microenvironment provides valuable insights and yields outputs that closely resemble actual physiological conditions.

## 3 Force-dependent molecular pathways in hypoxia

### 3.1 Oxygen concentration-dependent alterations of biochemical pathways

#### 3.1.1 Hypoxia-inducible factors

HIF is a transcription factor composed of an oxygen-sensitive α subunit and a heterodimeric β subunit that regulates angiogenesis to restore perfusion and oxygenation. Three HIF-α proteins (HIF-1α, HIF-2α, and HIF-3α) mediate the adaptive transcriptional response to hypoxia in both normal and tumor cells. (Acerbi et al., 2015). HIF-1α stimulates tumor metastasis to more oxygenated region by activating several oncogenic growth factors and increases the resistance of tumor cells to apoptosis by suppressing Bid and Bax, pro-apoptotic proteins of the Bcl-2 family, and enhancing the expression of apoptosis inhibitors, respectively (Erler et al., 2004). HIF-1 is a transcription factor that regulates the expression of numerous genes involved in adaptation and survival in oxygen-deficient environments (Harris et al., 1980; Storm et al., 2005; Choudhry and Harris, 2018). HIF-1α mediates the switch of glucose metabolism from oxidative phosphorylation to glycolysis in tumor cells, and hypoxia stimulates the Warburg effect (Koukourakis et al., 2016). The Warburg effect, also known as aerobic glycolysis refers to a characteristic metabolic change observed in cancer cells, where cancer cells prefer glycolysis that converts glucose to pyruvic acid, even in the presence of sufficient oxygen (Sahoo et al., 2022).

Like other HIF proteins, HIF-2 plays a central role in responding to changes in intracellular oxygen levels and orchestrating adaptive responses to hypoxic conditions. HIF-2 is composed of two major subunits, HIF-2α and HIF-1β, also known as aryl hydrocarbon receptor nuclear translocators (ARNTs). The HIF-2 signaling pathway is primarily regulated by the stability of the alpha subunit HIF-2α, which is sensitive to changes in oxygen levels. This pathway involves a series of molecular events that occur under hypoxic conditions to stabilize HIF-2α and initiate transcriptional responses. Under normal oxygen levels, normoxia, HIF Prolyl Hydroxylases (PHD) hydroxylates specific proline residues in HIF-2α, which marks HIF-2α for ubiquitin-mediated degradation via Von Hippel-Lindau (VHL), an E3 ubiquitin ligase complex (Chen W. et al., 2016).

HIF-2α stabilization prevents proline hydroxylation by inhibiting the activity of PHD under hypoxic conditions (Urrutia et al., 2016). This stabilization of HIF-2α prevents degradation and allows it to accumulate within cells. In heterodimer formation, stabilized HIF-2α heterodimerizes with HIF-1β/ARNT to form a functional HIF-2 complex. This complex translocates to the nucleus and binds to specific DNA sequences known as hormone response element (HRE) located within the promoters of target genes. Target gene transcription is activated when the HIF-2 complex binds to the HRE. These target genes include those involved in angiogenesis, erythropoiesis, glucose metabolism, and various processes that help cells adapt and survive hypoxic conditions (Chen et al., 2007). HIF-2 helps supply oxygen and nutrients to hypoxic tissues by regulating the expression of genes that promote angiogenesis, erythropoietin (EPO), and other erythropoiesis-related genes to that produce erythrocytes. However, it is also involved in erythrocyte production (Kobayashi et al., 2022).

**FIGURE 3:**
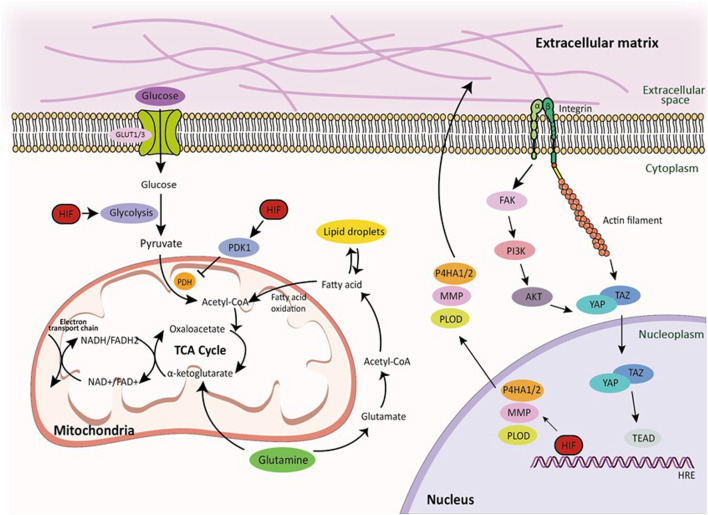
Glucose, lipid, and amino acid metabolism pathways and integrin-mediated mechanotransduction by HIF signaling. Transmembrane protein integrins serve as molecular linkage between the ECM in the extracellular space and cytoskeletal network in the intracellular cytoplasmic space, facilitating the transmission of mechanical forces induced by the actin retrograde flow via mechanosensitive focal adhesion proteins. Hypoxia directly regulates ECM composition through the enzymes. Furthermore, HIF signaling regulates glucose, lipid, and amino acid metabolic pathways in cellular metabolism under hypoxic conditions. This demonstrates how HIF signaling regulates cellular metabolism in hypoxia and highlights the tight relationship between these pathways and cancer progression. Within these pathways, the TCA cycle, glutamine metabolism, and NADH production play important roles, further highlighting the complex relationship between HIF signaling and cellular metabolism.

HIF regulates genes related to glucose metabolism, allowing cells to switch to glycolysis for energy production, through the Warburg effect, under hypoxia and can modulate cell growth and proliferation by regulating various signal transduction pathways (Seo et al., 2020) (Figure 3). Like most normal healthy cells, it depends on oxidative phosphorylation, i.e., aerobic respiration (Koppenol et al., 2011). The Warburg effect induces greater glucose uptake in cancer cells than in normal cells due to increased expression of glucose transporters (GLUT1) in the cell membrane of cancer cells, allowing the cancer cells to absorb more glucose from the bloodstream. Cancer cells preferentially rely on glycolysis to metabolize glucose, resulting in increased glycolytic activity even when sufficient oxygen is available to produce lactate as a by-product. Furthermore, mitochondrial function is often impaired, and oxidative phosphorylation is reduced. The mitochondria of these cells may be less efficient in producing energy via the citric acid cycle and electron transport chain. According to the Warburg effect, because glycolysis produces more energy than oxidative phosphorylation, cancer cells can rapidly produce ATP, which is essential for rapid growth and proliferation (Koppenol et al., 2011). The Warburg effect may help cancer cells adapt to the hypoxic and nutrient-poor TME. Reliance on glycolysis allows cells to generate energy even under hypoxia and limited nutrient availability. Switching to glycolysis can promote cell survival by inhibiting apoptosis (programmed cell death), which allows tumor cells to evade apoptotic signals (Zhao S. et al., 2021).

#### 3.1.2 HIFs and relevant transcription factors in tumor growth and metastasis

Hypoxia induces metabolic reprogramming, glycolysis, the TCA cycle, oxidative phosphorylation, fatty acid metabolism, and amino acid metabolism, altering cellular energy production and molecular synthesis (Table 2). HIFs regulate glucose metabolism in several ways. HIF activation under hypoxia occurs when HIF is stabilized and is activated when oxygen levels in the cellular environment drop due to the presence of diseases or impaired blood circulation. Under normoxic conditions, HIF rapidly breaks down. However, under hypoxic conditions, the degradation process is inhibited, and HIF accumulates in cells (Zheng et al., 2021). HIF activation stimulates the expression of enzymes involved in glycolysis, which is an early step in glucose metabolism that does not require oxygen. This allows cells to generate energy (ATP) from glucose, even under hypoxia. Enhanced glycolysis is particularly beneficial for rapidly dividing cells such as cancer cells. Cells switch from oxidative phosphorylation (oxygen-dependent) to anaerobic glycolysis (oxygen-independent) under hypoxic condition. Anaerobic glycolysis is less efficient in terms of ATP production but can provide energy even when oxygen is limited (Semenza, 2012).

**TABLE 2:** The effects of hypoxia on various metabolic pathways.

Metabolic pathway	Molecular mechanisms	Hypoxia effect	References
Glycolysis	HIF-1α (Hypoxia-Inducible Factor-1α) is stabilized and activates glycolytic enzymes like hexokinase and pyruvate kinase	Enhanced glycolytic activity to produce ATP in the absence of sufficient oxygen	Zheng et al. (2021)
Oxidative phosphorylation	Electron transport chain (ETC.) and ATP synthase activity are compromised	Impaired due to decreased oxygen as the terminal electron acceptor, reducing ATP synthesis	Shukla et al. (2017)
Fatty acid metabolism	Carnitine palmitoyltransferase I (CPT-I) and other enzymes involved in fatty acid oxidation are regulated by oxygen reduction	Shift from fatty acid oxidation to glycolysis for ATP production	Du et al. (2017b)
Amino acid metabolism	HIF-1α influences enzymes involved in amino acid metabolism	Altered amino acid utilization and metabolism, impacting protein synthesis and degradation	Bertero et al. (2019)
Citric acid cycle (TCA Cycle)	Enzymes of the TCA cycle are regulated by reduced mitochondrial respiration	Reduced activity due to limited oxygen availability, leading to decreased generation of reducing equivalents (NADH and FADH2)	Koukourakis et al. (2016)

HIF can increase the expression of glucose transporters such as GLUT1 in cell membranes (Harris et al., 1980; Sotgia et al., 2012). This allows cells to absorb more glucose from the extracellular environment, thereby ensuring an adequate supply for glycolysis. HIF-1α mediates the switch from oxidative phosphorylation to glycolysis in glucose metabolism in tumor cells, and hypoxia stimulates the Warburg effect. Because glycolysis produces much less ATP per glucose molecule than oxidative phosphorylation, cells require a sufficient amount of glucose to compensate for the high energy demand (Koppenol et al., 2011). HIF-1α upregulates the translocation of GLUT, such as GLUT1 and GLUT3, to the cell membrane, thereby promoting glucose uptake into tumor cells for glycolysis (Chan et al., 2011). GLUT1 and GLUT3 have been shown to be overexpressed in various types of cancer. HIF-1α can enhance glycolytic activity by regulating several glycolytic enzymes in tumor cells to convert glucose into glucose-6-phosphate. However, cells cannot export glucose-6-phosphate via GLUT; therefore, they donate glucose molecules for glycolysis.

HIF-1α inhibits the expression of pyruvate dehydrogenase (PDH) and regulates the conversion of pyruvate to acetyl CoA through the transcription of pyruvate dehydrogenase kinase 1 (PDK1) (Kim et al., 2006). HIF-1α further stimulates this conversion by upregulating lactate dehydrogenase (Bertero et al., 2019) which catalyzes the reaction (Liu et al., 2022), and anaerobic glycolysis results in the accumulation of lactate as a byproduct. Lactate can be used as a signaling molecule to trigger various responses, including angiogenesis and inflammation. As a metabolic adaptation, HIF induced changes in gene expression help cells adapt to hypoxia by promoting survival and minimizing damage. This may include altering the expression of metabolic enzymes and transporters to optimize energy production under hypoxic conditions. Under hypoxia, HIF activation supports energy production through enhanced glycolysis and regulates other cellular processes important for survival in an oxygen-poor environment (Wu et al., 2015).

Lipid metabolism constitutes various adaptive mechanisms to maintain cellular homeostasis and provide energy to ensure cell survival and function under hypoxic conditions. HIF is activated in response to hypoxia, where general degradation of HIF is inhibited, stabilized, and activated, translocates to the nucleus, binds to specific DNA sequences, and initiates transcription of target genes. HIF activation is associated with increased lipid accumulation, particularly in certain cell types, and may be a protective response to energy storage for future use when the availability of oxygen and glucose is limited (Lee et al., 2014).

HIF regulates adipogenesis by differentiating pre-adipocytes into mature adipocytes that determine the formation and function of the adipose tissue, which is the primary site of lipid storage in the body (Wang P. et al., 2022). Furthermore, HIF activation upregulates the expression of fatty acid transporters and enzymes involved in fatty acid oxidation, allowing cells to utilize fatty acids as alternative energy sources when glucose availability decreases. HIF promotes the formation of intracellular lipid droplets, which are structures that store excess lipids so that stored lipids can be mobilized and utilized for energy production when required. As HIF can regulate the genes involved in cholesterol metabolism, including those involved in cholesterol synthesis and absorption, it helps maintain cellular cholesterol homeostasis under hypoxic conditions. HIF activation can regulate sterol regulatory element-binding protein (SREBP) signaling, a key regulator of lipid synthesis and uptake (Choi et al., 2008). Specifically, hypoxia-mediated degradation of SREBP2 helps cells take up exogenous cholesterol, protecting them from statin-induced apoptosis. (Dickson et al., 2023). Lipid metabolism is regulated the expression of genes involved in adipogenesis. Due to the hypoxia-induced Warburg effect, the nutrients normally used for oxidative phosphorylation in ATP production are used for lipid and amino acid synthesis to promote tumor growth.

Mechanical signals from the ECM exert a profound impact on cellular processes such as proliferation, differentiation, and apoptosis. This influence is mediated by the regulation of various intracellular signaling pathways, including the Hippo signaling pathway and growth factors (Ramirez and Rifkin, 2003; Misra and Irvine, 2018). These intricate processes entail the metabolism of nutrients for both energy production and the biosynthesis of macromolecules. Particularly, extracellular mechanical cues have discernible effects on lipid metabolism (Romani et al., 2021). A soft ECM, reducing extracellular forces, triggers processes such as neutral lipid and cholesterol synthesis by influencing Golgi rheology. This, in turn, leads to the inactivation of Lipin-1, a critical molecular contributor to glycerolipid biosynthesis and gene regulation (Romani et al., 2019).

Lipid metabolism provides an alternative energy source when glucose levels are low; for example, triglycerides are converted to glycerol and fatty acids with the help of lipoprotein lipase (LPL). Acetyl-CoA production is enhanced by hypoxia through the upregulation of acetyl-CoA synthetase 2 (ACSS2) (Xu et al., 2014). Since ACSS2 is responsible for converting acetate to acetyl-CoA, TCA cycle entry is inhibited, and acetyl-CoA is used, among other things, to synthesize membrane phospholipids. ACSS2 expression is also associated with increased tumor aggressiveness in patients with cancer, and hypoxia-induced lipid metabolism reprogramming results in fatty acid accumulation, which, upon reoxidation, promotes tumor growth and survival (Schug et al., 2015). By profiling the proteins interacting with HIF-1α, fatty acid binding protein 5 (FABP5) was identified as an important HIF-1α binding partner. FABP5 enhances HIF-1α activity by promoting HIF-1α synthesis while disrupting the FIH/HIF-1α interaction. Based on confirmation in liver cancer cells, the upregulation of FABP5 by fatty acids induces cancer by reprogramming lipid metabolism via HIF-1 (Seo et al., 2020). Furthermore, HIF-1 and HIF-2 inhibit carnitine palmitoyltransferase 1A (CPT1A), reduce fatty acid transport into the mitochondria, and force fatty acids into lipid droplets for storage. Droplet formation occurs independently of the lipid source but only when CPT1A is inhibited. Functionally, the increased expression of CPT1A restricts tumor growth (Du et al., 2017b). Thus, lipid metabolism generates various signaling molecules, such as specific lipids and lipid-derived mediators.

HIF is involved in amino acid metabolism to support energy production, maintain redox balance, and regulate cellular signaling under hypoxic conditions. HIF activation under hypoxia is achieved by inhibiting the normal breakdown of HIF when oxygen levels decrease. Stabilized HIF then translocates to the nucleus, binds to specific DNA sequences, and initiates the transcription of target genes. Glutamine is a non-essential amino acid that is essential under certain conditions, including hypoxia. HIF upregulates the expression of genes involved in glutamine metabolism. Glutamine is utilized by cells for various purposes, including as a substrate in the TCA cycle to generate energy (Winkler et al.; Yoo et al., 2020). Glutamine is converted to glutamate by glutaminase to α-ketoglutarate or pyruvic acid, which is a substrate of the TCA cycle. The conversion of glutamate to pyruvic acid is known as glutaminolysis and occurs at high rates in cancer cells. Myc proto-oncogene protein (c-Myc) regulates glutamine uptake by upregulating glutamine transporters and increases the expression of glutaminase 1 (GLS1) (Ganguly et al., 2022).

Hypoxia plays a complex role in amino acid metabolism. Hypoxia induces a reduced TCA cycle in tumor-repopulating cells (TRCs), which hypoxia-induced reactive oxygen species (ROS) activate Akt and NF-κB (Winkler et al., 2020). Fumarate accumulates in the TCA cycle of hypoxic TRC, reduces glutathione succinylation and nicotinamide adenine dinucleotide phosphate (NADPH)/NADP + ratio, and increases ROS levels (Singhal et al., 2021). Mechanistically, hypoxia-enhanced HIF-1α transcriptionally downregulates mitochondrial phosphoenolpyruvate carboxykinase (PCK2) expression, leading to TCA cycle attenuation and fumarate accumulation (Tang et al., 2019).

HIF-2α promotes c-Myc activity and sensitizes cells to hypoxia-induced apoptosis. The basic helix-loop-helix leucine zipper (bHLH-LZ) transcription factor c-Myc directly regulates DNA replication and the expression of 10%–15% of all genes in the genome (Wernig et al., 2008). However, upregulation of c-Myc may help maintain a functional TCA cycle despite the Warburg effect and may provide nutrients (Wang H. et al., 2022; Lv et al., 2022).

HIF activation increases the expression of amino acid transporters, thereby facilitating the cellular uptake of amino acids and maintaining the supply of amino acids required for various cellular processes. HIF also regulates the expression of enzymes involved in amino acid catabolism, including those involved in the breakdown of certain amino acids for energy production and other metabolic pathways. Since amino acids play a role in maintaining the cellular redox balance, changes in HIF regulation of amino acid metabolism can produce ROS and overall oxidative stress within cells (Janbandhu et al., 2022). Some amino acids are precursors of signaling molecules, such as nitric oxide (NO) and polyamines, which are involved in various cellular processes, and HIF activation is involved in cellular signaling pathways (Taylor and Scholz, 2022). HIFs upregulate the expression of L-amino acid transporter 1 (LAT1), a transporter responsible for transporting of large neutral amino acids such as leucine and phenylalanine (Zhang et al., 2021). HIF-2α induces mammalian target of rapamycin complex 1 (mTORC1) activity by expressing the amino acid transporter LAT1 (Elorza et al., 2012). ASCT2, alanine, serine, and cysteine transporter 2 (ASCT2) are HIF regulated transporters that uptake glutamine and contribute to cellular glutamine availability under hypoxic conditions. HIF-2α triggers hypoxia-induced gene expression of ASCT2 (SLC1A5 variant), which mediates glutamine-induced ATP production, glutathione synthesis, and conferring gemcitabine (Yoo et al., 2020).

### 3.2 Hypoxia-induced mechanotransduction in 3D ECM

#### 3.2.1 Mechanotransduction by yes-associated protein (YAP)/transcriptional co-activator with PDZ-binding motif (TAZ) signaling

Yes-associated protein (YAP) and transcriptional co-activator with PDZ-binding motif (TAZ) regulate the transcription of the HIPPO tumor suppressor pathway and are used in tissue repair. YAP/TAZ acts as an extracellular signal transducer that converts tissue-level changes into intracellular biochemical signals in response to changes in the microenvironment and cellular architecture (Dupont et al., 2011).

**FIGURE 4:**
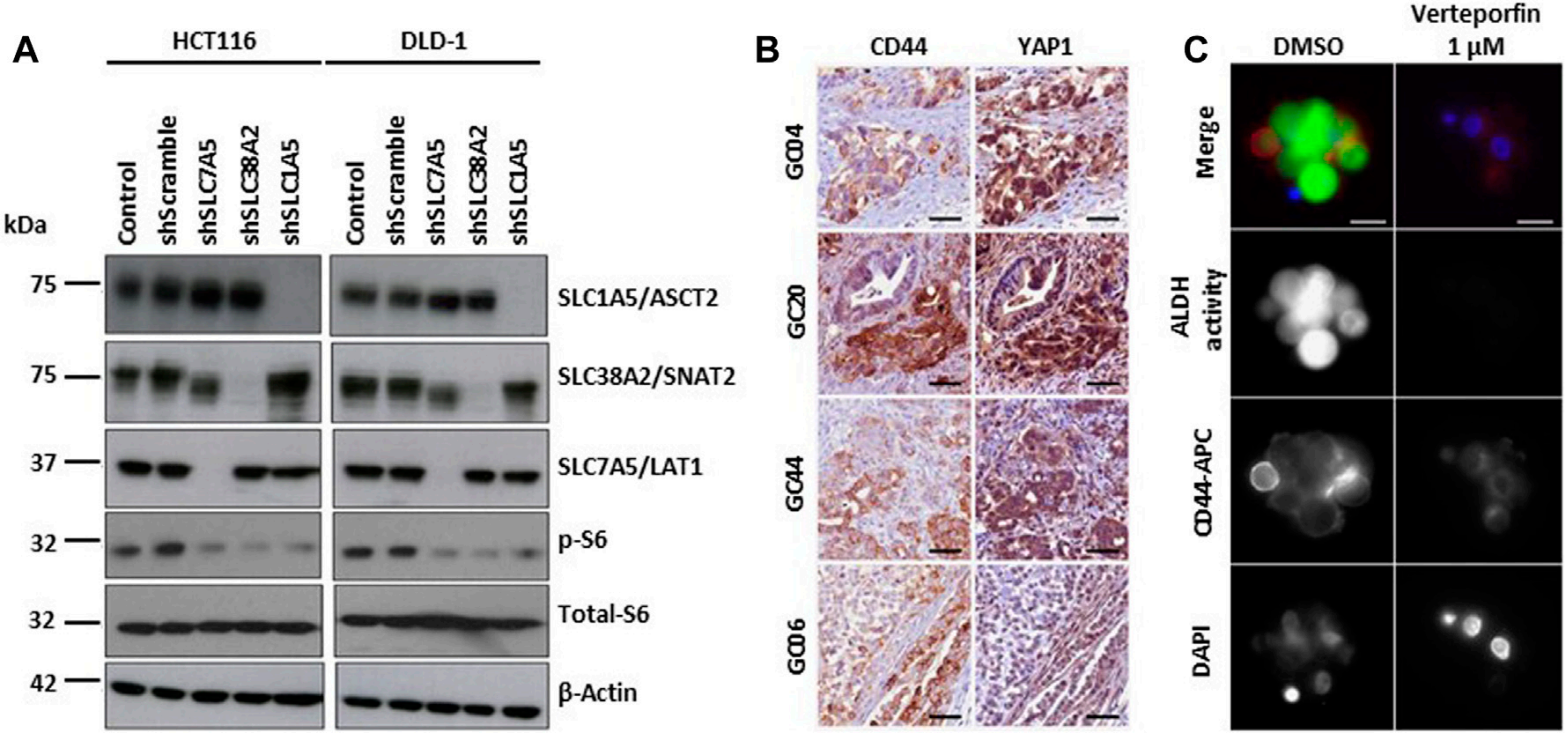
YAP/TAZ signaling pathways in hypoxia. **(A)** Depletion of amino acid transporters (AATs including SLC1A5/ASCT2, SLC7A5/LAT1, and SLC38A2/SNAT2) inhibits the activation S6 ribosomal protein (pS6), a downstream target of mTOR pathway. Representative western blot images show the changes in the protein expression of AATs, total- and phospho-S6 ribosomal protein in HCT116 and DLD-1 cells after the shRNA transfection. β-actin was used as protein loading control. Copyright obtained from reference [Kandasamy P et al., Molecular Oncology (2021)]. **(B)** Y/T-TEAD transcriptional activity is increased in CSC CD44-positive cells. Representative images of YAP1 and CD44 detection by immunohistochemistry performed on 3 μm serial tissue sections of human primary Gastric Cancer. GC20, GC04 and GC44 are intestinal histological subtype GC; GC06 is a diffuse type GC. Scale bar: 50 μm. Copyright obtained from reference [Giraud J, et al., International Journal of Cancer (2020)] **(C)** Verteporfin inhibits the expression of CSC markers in GC cell lines and patient-derived primary GC xenografts (PDX). Representative images of CD44 expression and ALDH activity determined by immunofluorescence analysis on live GC10-treated tumorspheres. CD44 staining, ALDH activity, and the nuclei were marked red (using anti-CD44-APC antibodies), green (using the ALDEFLUOR reagent), and blue (using DAPI), respectively. Scale bar: 10 μm. Copyright obtained from reference [Giraud J, et al., International Journal of Cancer (2020)].

YAP1/TAZ regulates amino acid metabolism by upregulating the expression of the amino acid transporters SNAT2, LAT1 and ASCT2. Increased amino acid uptake activates mTORC1, a key regulator of cell growth, and stimulates cell proliferation (Figure 4A) (Kandasamy et al., 2021). Inhibition of mTORC1 blocks YAP1/TAZ-mediated tumorigenesis (Park Y. Y. et al., 2016). YAP/TAZ are also associated with cancer stem cell (CSC) activity (Kim et al., 2018). In particular, gastric carcinoma is a heterogeneous tumor comprising clusters of chemoresistant cancer stem cells (CSCs), with subpopulations expressing elevated CD44 and high ALDH activity (Nguyen et al., 2017). YAP1 and TAZ oncoprotein (Y/T) interact with TEA domain family member 1 (TEAD) transcription factors to promote cell survival and proliferation in multiple tissues. Verteporfin effect inhibited Y/T-TEAD transcriptional activity, cell proliferation and CD44 expression, reduced the pool of tumor-forming CD44+/aldehyde dehydrogenase (ALDH)-high gastric CSCs, and inhibited GC tumor growth *in vivo* (Giraud et al., 2020) (Figures 4B,C).

YAP/TAZ activates CCN family oncogenes such as CCN family member CCN1 (Cyr61), also known as connective tissue growth factor (CTGF) and cysteine-rich angiogenesis inducer 1 (CYR1) (Xu et al., 2019). CCN proteins have been implicated in cardiovascular and skeletal development, injury repair, inflammation, and cancer development. They function by binding to integrin receptors or regulating the expression and activity of growth factors and cytokines (Kim et al., 2018). CCN1 (Cyr61) has been reported to inhibit YAP/TAZ activity by limiting its YAP/TAZ nuclear translocation, promoting the formation of adherent junctions, and stabilizing intercellular contacts (Cao et al., 2020). In contrast, luteolin causing a significant decrease in the expression of CTGF and CYR61 (Cao et al., 2020), inhibits the expression and nuclear translocation of YAP/TAZ and interferes with the transcriptional activity of YAP/TAZ (Cao et al., 2020). HIF induces the expression of CCN2 (CTGF) under hypoxic conditions. Hypoxia increases transcription by inducing HIF binding to the hypoxia response element within the CCN2 promoter. CCN2 plays an important role in fibrosis, angiogenesis, and tissue repair. CCN2 has also been shown to be upregulated in response to fibrotic cytokine-TGF-β and constitutively overexpressed in fibrotic conditions (Futakuchi et al., 2018). For example, CCN2 can inhibit angiogenesis when interacting with VEGF or stimulate it by interacting with TGF-β (Futakuchi et al., 2018).

#### 3.2.2 Modulation of ECM deposition in hypoxic condition

Excessive accumulation of ECM causes fibrosis and leads to stiffening of the ECM. Hypoxia is associated with ECM deposition. Fibroblast activation of the ECM is initiated by several tumor-released proteins and is also associated with hypoxia-induced TGF (Morimoto et al., 2021). Hypoxia directly regulates ECM composition through the enzymes P4HA1, P4HA2, and PLOD2 (Morimoto et al., 2021). Hydroxylation of proline and lysine residues, which are various post-translational modifications of the procollagen α-chain, improves the thermal stability of procollagen by hydroxylating proline to 4-hydroxyproline and allows procollagen to form a stable triple helix. HIF-1 activates expression of genes encoding collagen prolyl (P4HA1 and P4HA2) and lysyl (PLOD2) hydroxylases. P4HA1 and P4HA2 are required for collagen deposition, whereas PLOD2 is required for ECM stiffening and collagen fiber alignment (Gjaltema et al., 2016).

P4HA1, P4HA2, and PLOD2 mediate the remodeling of ECM composition, alignment, and mechanical properties in response to hypoxia. Collagen prolyl 4-hydroxylase (P4H) is an α2β2 tetrameric α-ketoglutarate (α-KG)-dependent dioxygenase that catalyzes 4-hydroxylation of proline to promote the formation of the collagen triple helix, thereby releasing succinate as a product. The P4H αsubunit (P4HA) is responsible for both peptide binding and catalytic activity (Kaluz et al., 2021). By modulating alpha-ketoglutarate (α-KG) and succinate levels, P4HA1 expression reduces proline hydroxylation on HIF-1α, enhancing its stability in cancer cells. Activation of the P4HA/HIF-1 axis enhances cancer cell stemness and is accompanied by decreased oxidative phosphorylation and ROS levels (Xiong et al., 2018). P4HA1 promotes chemoresistance by regulating HIF-1-dependent cancer cell lineage. Targeting collagen P4H inhibits tumor progression. Collagen is a major component of the ECM, and collagen crosslinking and deposition depend on lysyl hydroxylation catalyzed by procollagen-lysine, 2-oxoglutarate 5-dioxygenase (PLOD) (Gjaltema et al., 2016).

Abnormal lysyl hydroxylation and collagen crosslinking contribute to the progression of many collagen-related diseases, such as cancer. Three lysyl hydroxylases (LH1, LH2 and LH3) were identified and encoded by PLOD1, PLOD2, and PLOD3, respectively. Several cytokines, transcription factors, and miRNAs regulate PLOD expression. PLOD dysregulation promotes cancer progression and metastasis. PLOD1 hydroxylates lysines in the α-helical or central domain of procollagen, and PLOD2 is responsible for the hydroxylation of lysine residues in procollagen telopeptides. Both are transcriptionally activated by HIF-1α, but only PLOD2 activation can alter collagen crosslinking patterns (Du H. et al., 2017).

Extracellular proteolytic enzymes such as MMPs mediate changes in the microenvironment during tumor progression and are important contributors to tumor progression, invasion, and metastasis, including ECM degradation, basement membrane degradation, and angiogenesis promotion (Bonnans et al., 2014). It releases bioactive molecules and modulates cell signaling. MMPs belong to the family of zinc-dependent endopeptidases and are composed of zinc-binding catalytic domains that are essential for proteolytic activity that degrade various ECM components, including collagen, elastin, laminin, fibronectin, and proteoglycans. MMPs are synthesized as inactive zymogens (pre-enzymes) that must be proteolytically activated for their functionality.

MMPs can be activated by various mechanisms, including proteolytic cleavage by other enzymes (Visse and Nagase, 2003). Serine proteases or other MMPs cleave the zymogen prodomain, remove the inhibitory segment, and expose the catalytic sites for substrate binding and cleavage. Another activation mechanism involves modification of thiol groups in MMP molecules. This can occur through oxidation, often induced by ROS produced by leukocytes (Gabbia et al., 2021). ROS can modify the thiol groups in MMP structures, resulting in conformational changes that activate enzymes (Gabbia et al., 2021).

MMPs can break down ECM components, including collagen and proteoglycans, creating pathways for tumor cells to migrate through tissues and promote invasion (Munoz-Najar et al., 2006). The basement membrane provides a barrier for separating the epithelial and endothelial tissues. MMPs break down the basement membrane, allowing tumor cells to break through this barrier, invade the surrounding tissue, or enter the bloodstream for metastatic spread. Furthermore, MMPs are involved in angiogenesis. Breakdown of ECM components can stimulate the growth of new blood vessels into tumors and deliver nutrients and oxygen. MMPs release bioactive molecules from the ECM, including growth factors and cytokines, which can further support tumor cell growth, migration, and survival. MMPs cleave and release membrane-bound signaling molecules, thereby influencing cell signaling pathways (Stetler-Stevenson and Yu, 2001; Constance and Brinckerhoff, 2002). MMP-1 (collagenase-1) is known to have the ability to specifically degrade collagen type I and type III, which are major components of the ECM of connective tissues.

MMP1 is involved in tissue remodeling during development, wound healing, and diseases characterized by ECM degradation, such as rheumatoid arthritis and cancer metastasis (Mori et al., 2012). MMP-2 (gelatinase A) is primarily involved in the breakdown of denatured collagen (gelatin), including various ECM components, such as type IV and V collagen. MMP-2 is associated with angiogenesis, tissue remodeling, and tumor invasion, making it an important enzyme in cancer progression (Munoz-Najar et al., 2006). Stromelysin-1 (MMP-3) is involved in the degradation of various ECM components, including proteoglycans, fibronectin, laminin, and type IV collagen. It also activates other pro-MMPs. MMP-3 is involved in tissue repair, inflammation, and tumor invasion. MMP-7 (also known as Matrilysin) plays a role in degradation of a variety of substrates, including ECM components, proteoglycans and non-matrix molecules (Shantha Kumara et al., 2021). MMP-7 is involved in tissue remodeling during development, wound healing, and cancer progression and has also been implicated to play a role in inflammatory bowel diseases. Similar to MMP-2, MMP-9 (also known as gelatinase B) degrades gelatin and type IV collagen, and exosomal thrombospondin-1 (THBS1) binds to FAK and MMP-9 to prevent ECM stiffness-dependent cancer invasion. It plays a role in angiogenesis, tissue remodeling and tumor invasion (Kessenbrock et al., 2010; de Almeida et al., 2022).

#### 3.2.3 Amino acid metabolism regulated by YAP/TAZ signaling

The activation of integrins regulates amino acid metabolism through mechanotransduction pathways, such as FAK/PI3K/AKT and YAP/TAZ signaling. ECM stiffness regulates the expression of amino acid synthases, glutamine catabolism and amino acid transport (Li Z. et al., 2022). Akt partially modulates glucose transport by phosphorylating and activating phosphatidylinositol-3-phosphate-5, which fosters carrier protein insertion into the cell membrane (Bieler et al., 2007). The transcriptional regulators TAZ and YAP (TAZ/YAP) have been shown to be pro-tumorigenic factors that drive many oncogenic traits, including the induction of cell growth, resistance to apoptosis, and activation of processes that promote migration and invasion. Rescue experiments with glutamine-derived metabolites suggest an essential role for glutamate and α-ketoglutarate (AKG) in TAZ/YAP-driven cell growth in the absence of glutamine (Yang et al., 2018). Analysis of enzymes that mediate the conversion of glutamate to AKG shows that TAZ/YAP induced the expression of glutamic-oxaloacetic transaminase (GOT1) and phosphoserine aminotransferase (PSAT1). In both cancer cells and cancer-associated fibroblasts (CAFs), we found that ECM stiffening activates glycolysis and glutamine metabolism, thus coordinating non-essential amino acid flux within the tumor niche (Bertero et al., 2019). Specifically, we demonstrated metabolic crosstalk between CAF and cancer cells, where CAF-derived aspartate sustains cancer cell proliferation, while cancer cell-derived glutamate balanced the redox state of CAFs to promote ECM remodeling (Bertero et al., 2019). The regulation of YAP/TAZ in the rigid ECM is achieved through mechanical signaling by the Ras-related GTPase RAP2 and mediates cellular responses. At low stiffness, mitogen-activated protein kinase 4/6/7 (MAP4K4/6/7) and Rho GTPase-activating protein 29 (ARHGAP29) bind to active RAP2, resulting in LATS1/2 activation and YAP/TAZ (Meng et al., 2018).

## 4 Cancer progression in hypoxic tumor-microenvironment

### 4.1 Mechanosensitive tumorigenesis

#### 4.1.1 Tumor immunosuppression in response to mechanical stresses and hypoxia

Tumor immunosuppression refers to the strategies employed by tumors to elude and stifle the immune system. The immune response of cancer cells is intricately influenced by the tumor environment. Adjusting the physical attributes of the ECM orchestrates immune responses in cancer cells (Piersma et al., 2020). The immune reaction to cancer correlates with factors such as programmed death-ligand (PD-L) 1 and TGF-β signaling (Chakravarthy et al., 2018). The TGF-β family plays a crucial role in regulating various cellular functions, such as cell growth, differentiation, adhesion, migration, and apoptosis. It is essential for embryonic development, including germ layer specification and patterning (Drabsch and ten Dijke, 2012).

**FIGURE 5:**
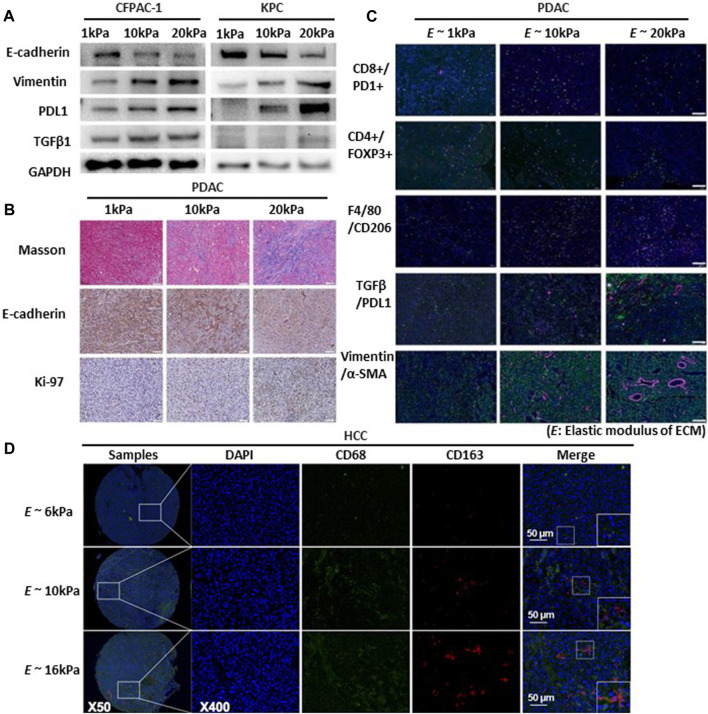
Substrate stiffness regulates tumor immunosuppression and progression. **(A)** EMT, TGFβ1, and PDL1 expression increased proportionally with the rise in matrix stiffness. Western blot detects the expression of EMT markers (E-cadherin, Vimentin), TGFβ1, and PDL1 in cystic fibrosis pancreatic adenocarcinoma cells (CFPAC) and KrasLSL-G12D/+; Trp53LSL-R172H/+; Pdx-1-Cre cells (KPC) cultured in 3D culture systems with varying matrix rigidity ranging from 1 to 20 kPa. Copyright obtained from reference [Zhang, Haoxiang, et al., Bioengineering and Translational Medicine (2023)]. **(B)** Multiplex IHC staining evaluating collagen production (Masson staining), EMT status (E-cadherin), and cell proliferation (Ki-67) in murine pancreatic ductal adenocarcinomas (PDAC) tumor tissues with varying degrees of matrix rigidity reveals that increasing ECM rigidity induces enhanced EMT and cell proliferation. Scale bar: 200 μm. Copyright obtained from reference [Zhang, Haoxiang, et al., Bioengineering and Translational Medicine (2023)]. **(C)** Extracellular matrix rigidity promotes immunosuppression by decreasing anti-tumor lymphocyte presence and upregulating presence of immunosuppressive cells and expression of marker proteins of the PDAC tumor. Using multiplex IF staining to map the TME of in different matrix rigidity murine PDAC tumors tissues include exhaust CD8^+^ cytotoxic T (CD8+PD1+), CD4^+^ T cells (CD4^+^), macrophages (F4/80+), M2 macrophages (F4/80+CD206+), TGFβ1/PDL1 expression and EMT status (Vimentin and α-SMA). Scale bar: 200 μm. Copyright obtained from reference [Zhang, Haoxiang, et al., Bioengineering and Translational Medicine (2023)]. **(D)** Increased matrix rigidity enhances the polarization of M2 macrophages in hepatocellular carcinoma cells (HCC). Two-color immunofluorescence staining for CD68 and CD163 (biomarkers for M2 macrophages) was performed on a tissue microarray obtained from buffalo rat models of HCC with varying liver stiffness backgrounds. Scale bar: 50 μm. Copyright obtained from reference [Xing, Xiaoxia, et al., The FEBS Journal 288.11 (2021)].

Substrate stiffness plays a crucial role in regulating the production of PD-L1, and F-actin formation is associated with the migration and spread of lung cancer cells (Miyazawa et al., 2018). Adhesion of head and neck squamous cell carcinoma (HNSCC) to the matrix is a crucial determinant of PD-L1 expression, which is mediated by a rigid matrix (Eichberger et al., 2020). Notably, the application of IFN-γ restored PD-L1 expression even in cells cultured on soft gels and under cell adhesion-free conditions. Moreover, in A549 cells—Lung cancer cells that do not inherently express PD-L1—IFN-γ triggers the production of PD-L1. This implies the existence of supplementary mechanisms beyond adhesion and mechanical cues in the regulation of PD-L1 production (Miyazawa et al., 2018). Considering the increased expression of PD-L1 on rigid substrates, further research is needed to explore the changes in immune factors resulting from mechanical forces. Furthermore, PD-L1 is an immune checkpoint protein that regulates immune responses. It is expressed on the surfaces of diverse cells, including cancer cells and immune cells such as macrophages (Dermani et al., 2019). Expression tumor immunosuppression-related marker protein (TGF-β1, and PD-L1) in PDAC cells increases with increased matrix stiffness (Figures 5A,B) (de Streel and Lucas, 2021). CD8^+^ T cells, also called cytotoxic T cells, activate CD4^+^ T cells. CD4^+^ T cells, also known as Helper T-cells, are integral components of the adaptive immune response (Bevan, 2004). In pancreatic cancer, higher ECM rigidity fosters immunosuppression by reducing the presence of antitumor lymphocytes (CD8^+^ T and CD4^+^ T cells) and increasing immunosuppressive cells (Tregs and CD206+ M2) within the high-rigidity group (Figure 5C) (Zhang et al., 2023).

Modulation of immune cells through mechanical stress holds promise for enhancing cancer immunotherapy. Immune cells (primarily T cells) respond strongly to applied physical forces and changes in the TME. Regulatory T cells (Tregs) represent a subtype of CD4^+^ T cells that suppress the activation and function of other immune cells (Kondelková et al., 2010). CD206+ M2 refers to M2 macrophages expressing the CD206 receptor. Increased matrix rigidity enhances the polarization of M2 macrophages, promoting their LOXL2 expression in hepatoceclluar carcinoma (HCC cell) (Xing et al., 2021) (Figure 5D). Deletion of this receptor diminishes TGF-β signaling, which in turn fosters recovery devoid of fibrosis (Nawaz et al., 2022). As mentioned above, immune function changes owing to the regulation of various immune factors according to the mechanical force in cancer. Cancer progression involves the transformation of the ECM (Walker et al., 2018). The extent of ECM stiffness alteration varies depending on the breast cancer subtype and its progression stage (Acerbi et al., 2015). Luminal breast cancer is the predominant breast cancer subtype and is often identified as hormone receptor-positive (HR-positive) breast cancer (Ignatiadis and Sotiriou, 2013). Luminal breast cancers exhibit reduced ECM remodeling and stiffness, consistently presenting with lower levels of immune infiltration and signals promoting invasion (Acerbi et al., 2015). As cancer cells respond to mechanical stress through mechanotransduction, targeting the ECM and its related functions could potentially offer effective strategies against breast cancer.

In particular, aggressive tumors and ECM stiffening are related, and this relationship differs depending on the breast cancer type. Among the breast cancer subtypes, basal-like and human epidermal growth factor receptor 2 (HER2)-positive tumor subtypes are characterized by heightened aggressiveness (Livasy et al., 2006). The elevated aggressiveness observed in certain breast cancers is associated with increased levels of infiltrating macrophages (CD68^+^ cells) and immune cells (CD45^+^ cells), contributing to increased collagen accumulation through stromal TGF-β signaling (Acerbi et al., 2015). These connections between tissue mechanics and inflammation synergistically enhance the aggressive nature of breast tumors.

As cancer cells grow, a hypoxic TME emerges. Tumor hypoxia influences cancer cell proliferation, migration, and the immune system by releasing various transcription factors and signaling pathways (Pennacchietti et al., 2003; Hill et al., 2009). The HIF family plays a critical role in the cellular response to hypoxia (Table 3). Moreover, HIF-1α is increased in oral squamous cell carcinoma (OSCC), controlling aggressiveness and tumor size (Sumera et al., 2023). In hypoxic conditions, the expression of HIF-α regulates CD4^+^ effector T cells, leading to increased immune suppression (Westendorf et al., 2017). The expression of HIF-1α serves as a prognostic indicator for patients with OSCC (Eckert et al., 2012). Gamma delta T cells (γδ T cells) differ from conventional αβ T cells in their T cell receptor (TCR) composition, as they express a distinctive TCR made up of γ and δ chains instead of the typical α and β chains (Deseke et al., 2020). Using the γδ T cells in cancer immunotherapy is a promising method to improve cancer treatment outcomes (Mensurado et al., 2023). However, under hypoxia, γδ T cells exhibit a reduced capacity for lysing ovarian cancer cells and downregulate calcium efflux, indicating a dampening of their cytotoxic effector functions (Sureshbabu et al., 2020).

**TABLE 3:** HIF signaling-related effects in different cancers.

Organ systems	Cell type	Alteration in cancer	References
Mouth	Oral squamous carcinoma	Tumor size increase	Sumera et al. (2023)
	Oral cancer	Cytotoxicity γδT cells expression increase	Sureshbabu et al. (2020)
Breast	Triple-negative breast cancer	miR-494 expression decrease	Li et al. (2022a)
	Triple-negative breast cancer	CDK13 expression increase	Lu et al. (2022)
Lung	Lung adenocarcinoma	PI3K/Akt and ERK pathway activation	Lee et al. (2006)
Pancreas	Pancreatic ductal adenocarcinoma	Glut1 and aldolase A mRNAs expression increase	Akakura et al. (2001)
	Pancreatic ductal adenocarcinoma	Prolyl 4-hydroxylase subunit alpha 1 (P4HA1) expression increase	Cao et al. (2019)
	Pancreatic ductal adenocarcinoma	TWIST expression increase	Chen et al. (2016a)
	Human pancreatic cancer	Leptin receptors (Ob-R) activation	Ren et al. (2014)

In triple-negative breast cancer (TNBC), hypoxia suppresses immune effector gene expression and decreases responsiveness to immunotherapy (Ma et al., 2022). This effect is mediated by HIF-1α. Triple-negative breast cancers are characterized by the absence of estrogen receptor (ER), progesterone receptor (PR), and HER2 expression (Foulkes et al., 2010). Diminished expression of immune-related genes in TNBC results in epigenetic suppression of effector genes and subsequent immune dysfunction of T cells (Ma et al., 2022). Moreover, increased HIF-1α in breast cancer regulates metastasis by specifically activating genes encoding various members of the LOX family (LOX/LOXL proteins) (Wong et al., 2011), which are involved in promoting tumor progression and suppressing immune responses in the TME. However, the function of the LOX family in tumors is controversial, as they can act as both tumor suppressors and metastasis promoters (Barker et al., 2012).

Under hypoxic conditions, a growth factor receptor/PI3K/Akt cascade (which includes Src, Ras, and mitogen-activated protein kinase) is activated, which is well known for its role in cell survival signaling pathways (Chen et al., 2001). It is tightly regulated and acts on the intracellular pathways in cancers (Rascio et al., 2021). In A549 cells, a lung cancer cell line, the PI3K/Akt pathway is activated under hypoxic conditions. When this pathway is blocked, cells exhibit resistance to apoptosis induced by UV light and etoposide under hypoxic conditions (Lee et al., 2006). L3.6pl cells, a pancreatic cell line, show resistance to gemcitabine-induced apoptosis under hypoxia. This resistance is associated with heightened phosphorylation and activation of protein kinase B (AKT), p38 mitogen-activated protein kinase (MAPK), extracellular signal regulatory kinase (Erk), and nuclear factor kappa B (NF- κB), which are signaling pathways associated with immune response (Yokoi and Fidler, 2004; Harikrishnan et al., 2018). Furthermore, many genes associated with hypoxia and the immune system have been identified within the realm of head and neck squamous cell carcinoma (HNSCC) (Bajrai et al., 2021). By studying immune-associated pathways and their associated genes, we can gain insights into how hypoxia and immune response contribute to the growth, invasion, metastasis, and overall aggressiveness of oral cancers.

Pancreatic stellate cells (PSCs) reside in the pancreas and play a crucial role in its pathogenesis. Once activated, PSCs play a central role in chronic pancreatitis and pancreatic cancer (Zhou et al., 2019). They contribute to the formation of a dense fibrotic stroma by secreting factors that promote cancer cell proliferation, angiogenesis, and immunosuppression (Vonlaufen et al., 2008). Moreover, in response to hypoxic conditions, PSCs demonstrate increased migration, upregulated expression of type I collagen, and increased production of VEGF. These pro-fibrogenic and pro-angiogenic roles which lead to the development of pancreatic fibrosis are involved in the pathogenesis of chronic pancreatitis and pancreatic cancer (Masamune et al., 2008). When exposed to hypoxia, pancreatic cancer cells elevate the expression of HIF-1α, GLUT1, and aldolase A, which are crucial for conferring resistance to apoptosis (Akakura et al., 2001). GLUT1 functions as a crucial facilitator of glucose transport, and its overexpression has been reported in malignant cancers (Carvalho et al., 2011). HIF binds to sites within the genetic code for aldolase A (ALDA) (Semenza et al., 1996). The increased presence of these components fosters anaerobic metabolism and increases cell viability under hypoxic conditions (Akakura et al., 2001). CAFs occupying a majority of the TME actively contribute to the heightened aggressiveness of pancreatic cancer (Matsuo et al., 2004). When exposed to hypoxic conditions, these fibroblasts undergo activation into an inflammatory state, triggered by interleukin-1α (IL-1α) released by pancreatic cancer cells (Matsuo et al., 2009). The secretion of IL-1α by the cancer cells enhances the metastatic potential, immune evasion capabilities, and proliferation rate in pancreatic cancer (Matsuo et al., 2004; Matsuo et al., 2009; Melisi et al., 2009; Xu et al., 2010).

#### 4.1.2 Enhanced malignancy of cancers

Cancer manifests aggressive and invasive behaviors including genetic mutations, TME, epithelial-mesenchymal transition (EMT), and evasion of the immune response. By interacting with ECM receptors, cancer cells perceive the stiffness of the surrounding ECM (Ingber, 2003). The malignancy of several tumors (e.g., breast, lung, and pancreatic cancers) is regulated by ECM stiffness (Haage and Schneider, 2015; Wei et al., 2015; Zhao D. et al., 2018).

Pancreatic cancer has a high potential for spreading metastasis to other organs. Proliferation and migration of pancreatic ductal adenocarcinoma (PDAC) cells are enhanced through upregulating various signaling pathways, such as the Wnt signaling pathway, Hippo signaling pathway, PI3K/Akt signaling pathway, EMT, and TGFβ signaling, leading to increased matrix stiffness (Zhang et al., 2023). Mechanical stress interacts with various biochemical signaling pathways that regulate cancer progression. Therefore, the development of targeted therapies and interventions is crucial.

Pancreatic cancer cells degrade the TME via MMPs (Bloomston et al., 2002). MMPs are a family of enzymes that break down ECM components (Kessenbrock et al., 2010). In Panc-1 cells, MMP expression of MMPs escalates under conditions of high stiffness, and this heightened expression is linked to increased tumor invasion (Maatta et al., 2000; Haage and Schneider, 2015). In breast cancer, the expression of MMP1, MMP-3, MMP-9, and MMP-13 contributes to the acquisition of invasive properties (Balduyck et al., 2000). Cancer is a complex disease, and the impact of changes in MMP levels can vary depending on the specific context and alternations involved during pancreatic cancer development (Ellenrieder et al., 2000). EMT involves specific alterations in marker proteins and cellular morphology (Brabletz et al., 2018). These changes progressed towards mesenchymal behavior when pancreatic cancer cell lines were cultivated on progressively rigid gels (Rice et al., 2017; Deng et al., 2022). The responsiveness of EMT induction to matrix stiffness is linked to the initial phenotype of cells. The BxPC-3 cell line, which is predominantly epithelial in nature, displayed heightened sensitivity, whereas the Suit2-007 cell line, which is predominantly mesenchymal, exhibited reduced sensitivity. The AsPC-1 cell line, which is characterized by an intermediate phenotype, demonstrated intermediate receptiveness (Rice et al., 2017). Depending on the cell type, the gradual transition observed *in vitro* underscores the adaptability of EMT.

Poor prognosis and malignancy of the diseased liver are associated with increased liver stiffness; therefore, many studies have analyzed the relationship between liver cancer and changes in the ECM composition (Xia et al., 2020). In hepatocellular carcinoma (HCC), cells placed on medium and stiff hydrogels adopt elongated and extended morphologies. Piezo1, a mechanosensitive ion channel, is upregulated at a high matrix stiffness (Zhao Q. et al., 2018). Piezo1 knockdown inhibits the proliferation of HCC and angiogenesis during elevated liver stiffness (Li M. et al., 2022). Notably, the stem-like phenotype of HCCLM3 and Huh7 cells, which are HCC cell lines, is governed by the matrix stiffness present within HCC tissues through the integrin-YAP pathway. While the levels of YAP expression remain unchanged, ANKRD and Connective tissue growth factor (CTGF) exhibited increased expression in response to increased matrix stiffness (Wei et al., 2022). The ankyrin repeat domain (ANKRD) family encodes a group of proteins involved in protein localization and signaling and CTGF is also involved in tissue repair, wound healing, and fibrosis (Lipson et al., 2012; Tanno et al., 2012). Consequently, altered gene expression regulated by a rigid substrate influences cancer progression. Furthermore, increased homeobox protein NANOG and octamer-binding transcription factor 4 (OCT4) transcription on a stiff matrix fosters YAP aggregation in the nucleus (Wei et al., 2022). HCC cells secrete more exosomes due to the action of Akt in the stiff ECM, which increases tumor growth (Wu et al., 2023). Additionally, in HepG2 cells, a hepatocellular carcinoma cell line, higher substrate stiffness amplifies nuclear paraspeckle assembly transcript 1 (NEAT1) expression, which activates the EMT process and cell proliferation by activating the WNT/β-catenin signaling pathway (Xu et al., 2021). Excessive activation of β-catenin signaling can promote aberrant cell growth and play a role in tumor development. Moreover, many types of cancers have been linked to dysregulated Wnt signaling, which regulates various biological conditions (Clevers and Nusse, 2012). These mechanisms in pancreatic cancer upregulate various facets of cancer cell proliferation.

Breast cancer is the most common cancer in women with the high mortality (DeSantis et al., 2019). Studies on breast cancer in a stiff ECM explore the impact of the physical properties of the TME on breast cancer progression and behavior. As breast tumors grow and progress, changes occur in the composition and organization of the ECM, including collagen fibers (Oskarsson, 2013; Insua-Rodríguez and Oskarsson, 2016). These modified mechanical properties of the ECM can activate specific signaling pathways that foster tumor cell survival and division, ultimately contributing to breast cancer growth.

Twist family bHLH transcription factor 1 (TWIST1), a basic helix-loop-helix (BHLH) transcription factor, plays a pivotal role in activating the EMT and promoting tumor invasion (Yang et al., 2004). In particular, enhanced matrix stiffness facilitates the nuclear translocation of TWIST1 by disengaging TWIST1 from its cytoplasmic binding partner G3BP stress granule assembly factor 2 (G3BP2), the TWIST1-G3BP2 mechanotransduction pathway (Wei et al., 2015). As breast cancer advances, the ECM undergoes increased stiffness and density owing to the deposition and crosslinking of collagen fibers (Colpaert et al., 2001; Conklin et al., 2011). Both increased matrix stiffness and G3BP2 downregulation are associated with poor survival in patients with breast cancer (Wei et al., 2015). Increased breast density linked to collagen I on mammograms is associated with a 4- to 6-fold higher risk of breast cancer (Guo et al., 2001). This study demonstrated that a collagen-rich environment alters the metabolism of breast cancer cells, leading to reduced glucose utilization and increased use of another carbon source, such as glutamine, for aerobic respiration (Morris et al., 2016).

The change in ECM stiffness is an important feature of the TME. Integrins sense mechanical stress in the ECM, and cancer cells can perceive and respond to mechanical stress in the TME through integrin receptors (Ingber, 2003; Levental et al., 2009)The molecular forces formed by the binding of integrins and ligands regulate cellular functions. To modulate the specific force delivered by the integrins, a tension gauge tether (TGT) was prepared using an oligonucleotide (Wang and Ha, 2013). The TGT method has shown that the adhesion and invadopodia maturation of metastatic cancer cells necessitate the presence of a particular integrin type, namely, α5β1 integrin, along with integrin tension exceeding 40pN (Kim et al., 2022). Furthermore, a micro-confinement TGT (µC-TGT) has been developed to comprehend alterations in integrin-mediated force during cell migration and adhesion within restricted microchannels. Elevated integrin tension and reduced confinement induce mesenchymal migration of breast cells (Kim et al., 2023). These findings indicate the influence of pico Newton forces transmitted through integrins on the regulation of cancer cell proliferation and metastasis.

### 4.2 Shear stress-induced alteration of cancer mechanotransduction in hypoxia

Cancer cells experience shear stress within the blood and lymphatic vessels. Several studies have shown that shear stress can alter cancer cell behavior (Lee et al., 2017). However, cancer is a heterogeneous disease, and the response to shear-induced alterations in mechanotransduction varies depending on the specific type of cancer (Lv et al., 2022).

Circulating tumor cells (CTCs) are cancer cells that originate from primary tumors and travel through the bloodstream, potentially leading to metastasis. The detection and analysis of CTCs has been the subject of extensive research (Cabel et al., 2017).

The EMT states of CTCs regulate the biological capabilities of cancer cells and have been shown to augment the EMT in breast cancer, involving the mechanotransducer YAP (Zhao B. et al., 2021). YAP can directly control signaling molecules associated with EMT. In breast cancer tissue samples, it has been observed that heightened expression of YAP target genes leads to the activation of TGF-β pathway, highlighting YAP’s regulatory role in TGF-β-dependent EMT (Pseftogas et al., 2020). These changes enhance the ability of CTCs to withstand shear stress stimuli in the bloodstream (Cao et al., 2016).

Under low shear stress conditions (2 dyn/cm^2^), MDA-MB-231 cells, a human breast carcinoma cell line, developed resistance to anoikis, characterized by increased Cav-1 protein expression (Li et al., 2019). Cav-1 is recognized as a tumor promoter in breast cancer, although its role in cancer is context-dependent and governs cancer cell metabolism through diverse molecular pathways (Sotgia et al., 2012; Gupta et al., 2014). The c-subunit of catalytic portion F1 and a proton channel (F1FO) ATP synthase refers to a specific subunit of the ATP synthase enzyme complex that provides the cell with an energy supply for cellular processes (Bonora et al., 2013). Furthermore, the c-subunit plays a vital role in stress-induced permeabilization of the inner mitochondrial membrane (IMM) through a calcium-induced permeability transition (Amodeo et al., 2021). In particular, FSS (fluid shear stress) increases the proliferation of MCF7 and MDA-MB-231 cells by reducing F1FO ATP synthase function (Park et al., 2022).

The effect of shear stress on prostate cancer varies depending on the type of cancer. In the context of metastasis, DU145 cells that have experienced fluid shear stress (FSS) exhibit strong resistance and possess effective cell-membrane repair capabilities (Hope et al., 2021). Similarly, resistance to FSS differed between SW620 (colorectal cancer cells found in lymph nodes) and SW480 (primary colorectal cancer cells). The heightened resistance of SW620 cells is closely associated with the downregulation of Piezo1 expression (Greenlee et al., 2022). Piezo1 is a mechanosensitive ion channel found on the cell membrane that interacts with membrane lipids (Cox et al., 2018). The reduced Piezo1 expression in metastatic SW630 cells suggests that the potential downregulation of Piezo1 is a survival mechanism for cancer cells during the transitional phase (Greenlee et al., 2022).

Hyaluronan or hyaluronic acid (HA) is a substance found in abundance in the ECM. Its production is increased in proliferating cells (Laurent and Fraser, 1992). HA binds to CD44, a cell surface receptor, and this enhances the migration, proliferation, and invasion of cancer cells (Cortes-Dericks and Schmid, 2017; Senbanjo and Chellaiah, 2017). Shear stress regulates the CD44/HA interaction, influencing cell rolling and the adhesion of CD44^+^ leukemic and hematopoietic progenitor cells. Specifically, only CD44^+^ cell lines (KG-1a and HL-60) exhibited high attachment under a shear stress of 0.2 dyn/cm^2^ (Christophis et al., 2011). This modification of cell rolling and adhesion under shear stress is regulated by selectins, which constitute the initial step in recruiting immune cells to the sites of inflammation (Rossiter et al., 1997). Additionally, alterations in the interactions between ligands and adhesive receptors in the blood and lymphatic vessels activate circulating T cells and regulate outside-in signaling (Chen and Zhu, 2013; Rossy et al., 2018).

Shear stress may interact with alterations in HIF, enabling cancer cells to infiltrate and invade surrounding tissues. Hypoxic conditions promote cancer cell migration and proliferation by activating various signaling pathways and the transcriptional activator HIF-1 (Semenza, 2001). This fluid-induced change in HIF-1α promotes NADPH Oxidase 4 (NOX4)-dependent ROS, regulating endothelial cell metabolism (Wu et al., 2017). NOX4 also plays a role in promoting inflammation, as its deletion leads to decreased H_2_O_2_ production and pro-inflammatory markers (Gray et al., 2016). The role of shear stress in cancer progression under hypoxic conditions remains relatively understudied, and further investigation is needed to understand its implications for tumor behavior and metastasis comprehensively.

## 5 Future perspectives

Cancer remains a global health challenge, and understanding the mechanisms that drive its progression is crucial for developing effective treatments. Hypoxia, accompanied by tumor metastasis, plays a critical role in promoting cancer cell invasion of neighboring matrices. As rapid tumor growth outpaces angiogenesis, leading to areas within the tumor that lack adequate oxygen supply, the hypoxic extracellular microenvironment is a key driver of cancer progression and metastasis. Recent studies have shown that hypoxia triggers a cascade of molecular events that enhances cancer cell migration, invasion, and resistance to therapy. Moreover, alterations in the ECM, including matrix viscoelasticity, extracellular fluid viscosity, ligand density, osmotic pressure, and oxygen concentration, have been recognized as critical determinants influencing cancer growth, progression, and metastasis.

Investigating the physical aspects regulating cancer cell migration under hypoxic conditions has several clinical implications. First, it offers potential therapeutic targets for inhibiting metastasis, which is responsible for most cancer-related deaths. Targeting the mechanical adaptation of cancer cells to hypoxia may help develop novel treatment strategies. Understanding the mechanical properties of cancer cells can aid in developing diagnostic tools. Researchers are exploring methods to detect cancer cells in the circulation (liquid biopsy) based on their mechanical properties, potentially offering a less invasive and more sensitive method for early cancer detection.

To advance the development of targeted cancer therapies, cellular mechanics, which deals with how cells interact with and respond to their physical environments, have emerged as a critical determinant to predict cancer migration under hypoxic conditions. The mechanical properties of cancer cells, such as elasticity, adhesion, and motility, and those of the ECM, such as matrix density, porosity, rigidity, and fiber alignment, are profoundly regulated by a hypoxic microenvironment. The intricate interplay of events establishes the mechanical properties of cancer cells in such a complex manner that targeting a singular regulatory step is likely to prove ineffective. Given the dependency of cancer mechanobiology, the development of sophisticated *in vitro* and *in vivo* experimental models and tools is imperative for comprehensive understanding. Therefore, understanding the interactions between cell mechanics and signaling pathways is crucial for developing targeted therapies to inhibit cancer cell movement under hypoxic conditions.
